# The Effects of Alloying Elements Cr, Al, and Si on Oxidation Behaviors of Ni-Based Superalloys

**DOI:** 10.3390/ma15207352

**Published:** 2022-10-20

**Authors:** Suyu Ma, Qingqing Ding, Xiao Wei, Ze Zhang, Hongbin Bei

**Affiliations:** 1 School of Materials Science and Engineering, Zhejiang University, Hangzhou 310027, China; 2 Polytechnic Institute, Zhejiang University, Hangzhou 310027, China

**Keywords:** Ni-based superalloys, oxidation behaviors, alloying effects, microstructures, oxidation resistance

## Abstract

Oxidation behaviors of three Ni-based model alloys and pure Ni in the temperature range of 700–1200 °C are investigated to reveal effects of Cr, Al, and Si on the oxidation resistance of Ni-based superalloys. The formation and integrity of consecutive chromia or alumina layers are important for excellent oxidation resistance. The addition of 20 at.% Cr can effectively improve the oxidation resistance of Ni-based alloys by forming a thin chromia film below 1000 °C, while adding 15 at.% Al has a beneficial effect on the oxidation resistance of Ni-based alloys at temperatures above 900 °C. The addition of 2 at.% Si to Ni-Al alloy is insufficient to form a protective SiO_2_ layer but can accelerate the formation of alumina, which enables Ni-Al alloy to form a consecutive inner alumina layer at a relatively low temperature of 800 °C and further improve the oxidation resistance above 800 °C.

## 1. Introduction

Ni-based superalloys have been widely used as high-temperature materials for applications such as boiler tubes and furnace flame hoods [1]. Under such tough service environments, oxidation resistance is one of the foremost requirements for the stability and reliability of these alloys, since oxidation can result in dealloying corrosion, loss of surface strength, crack initiation, and ultimately failure [2,3,4,5,6]. Therefore, investigating the oxidation performance of Ni-based superalloys is essential to provide insight for high-temperature applications.

The essence of the oxidation process is the selective oxidation reaction of specific elements. Because oxides separate oxygen from the base metal, one or both reactants must diffuse through the oxide scale to ensure that the reaction can continue [5]. Whether the oxide scale can effectively inhibit diffusion determines the oxidation resistance of the alloy. In an ideal case, the protective oxide scale should be highly stable, continuous, slow growing, free from cracks or pores, and dense [6]. However, among as many as 12 or 13 alloying elements in commercial Ni-based alloys, only Cr, Al, and Si can form thermal stable oxide phases, which meet conditions for good oxidation resistance [6,7,8,9]. According to the Ellingham/Richardson diagrams [2,5], the standard free energies of formation (ΔG) for Cr_2_O_3_, Al_2_O_3_, SiO_2_, and some alkali earth metal oxides are relatively low at the certain same temperature, which means that these oxides possess relatively high stability. However, the Pilling–Bedworth ratios (PBR) of these alkali earth metal elements are lower than 1, so they are theoretically impossible to form non-porous oxides [2,5]. Therefore, from the perspective of alloy composition, only Cr, Al, and Si are considered to have a crucial impact on the oxidation resistance of Ni-based superalloys. Meanwhile, the oxidation reaction strongly depends on thermodynamics and kinetics, implying that temperature is an important influencing factor. Consequently, it is necessary to explore the effects of Cr, Al, and Si on oxidation behaviors at different temperatures to lay the groundwork for understanding the oxidation of Ni-based superalloys.

Previous studies show that protective scales formed by Cr and Al are highly dependent on temperature. Chromia-forming alloys can form a continuous and compact Cr_2_O_3_ scale to provide excellent oxidation resistance at service temperatures not exceeding 900 °C. Their use at temperatures higher than 1050 °C is limited due to the formation of volatile CrO_3_ [10,11,12,13]. The alumina-forming alloys are commonly employed for practical applications, due to the slow growth rate and long-term chemical stability of Al_2_O_3_ above 800 °C [4,14,15,16,17,18,19]. Although there are some studies on the oxidation of binary or ternary alloys containing Al below 800 °C [20,21,22,23,24,25,26], the effect of Al on oxidation behaviors of Ni-based superalloys at low temperatures is relatively limited. As a minor element in Ni-based superalloys, Si can form a synergistic effect with Al to improve oxidation resistance. Yeh et al. [27] and Sato et al. [28] both report that the addition of Si in high-generation Ni-based single-crystal superalloys can accelerate the formation of Al_2_O_3_ to improve oxidation resistance at 1100 °C. Another benefit of this synergistic effect is reported by Wang et. al. [8], indicating that Si addition can enhance the adhesion between the base metal and Al_2_O_3_ layer in a Ni-15Cr-5Al-3Si model alloy at 1100 °C.

However, the composition of most commercial alloys used in previous studies is too complicated to exclude the influence of other alloying elements. The studies using simple model alloys lack a systematic comparison of these three elements at different temperatures. Thus, comprehensive studies using suitable binary or ternary alloy systems over a wide temperature range are desired. Here, two binary Ni-based model alloys (Ni-Cr and Ni-Al) are compared with Ni to study the effects of Al and Cr on oxidation resistance. Then, a ternary Ni-based model alloy Ni-Al-Si makes a comparison with the Ni-Al alloy to show the influence of Si. In a wide temperature range of 700 °C to 1200 °C, isothermal oxidation experiments up to 576 h are carried out. Subsequently, microstructures of oxide scales are characterized by advanced microscopy and the oxidation mechanism is discussed. This work might provide the scientific basis for the design of Ni-based superalloys with excellent oxidation resistance.

## 2. Materials and Methods

### 2.1. Materials

Three model Ni-based solid-solution alloys with the nominal composition shown in Table 1 were prepared by arc melting appropriate amounts of high-purity metals (>99.9 wt.%) in a water-chilled copper crucible under a partial Ar atmosphere. The arc-melted alloys were then drop-cast into a copper mold with a dimension of 12.5 × 12.5 × 120 mm^3^. The total weight loss of each alloy after drop casting was less than 0.1%.

Drop-cast bars were cold-rolled along the longitudinal ingot direction to a total thickness reduction of ~84%. A Ni plate was cold-rolled to a total thickness reduction of ~65%. The four cold-rolled plates were annealed at 1150 °C for 1 h and then cooled in air to obtain a fully recrystallized microstructure.

### 2.2. Isothermal Oxidation

Individual samples (10 × 10 × 2 mm^3^) were cut from heat-treated plates through electron discharge machining and ground down to 1200-grit SiC papers to ensure a flat and shiny surface. Before oxidation experiments, samples were cleaned successively with water, ethanol, and acetone in an ultrasonic cleaner and blow-dried immediately. Edge lengths of samples were measured by a caliper with an accuracy of 0.01 mm to calculate the surface area.

Isothermal oxidation experiments were carried out in static air at 700 °C, 800 °C, 900 °C, 1000 °C, 1100 °C, and 1200 °C using a muffle furnace. Alumina crucibles used in oxidation were heated at 1250 °C for 24 h to completely remove water and volatile substances. Oxidation kinetic curves were determined by using the static weight gain method. Samples were removed from the furnace at specific time intervals, cooled in air, and then weighed with an electronic balance with a resolution of 0.1 mg. Three specimens were simultaneously tested at each temperature for reproducibility of the data.

### 2.3. Microstructure Characterization

Specimens for cross-sectional analysis were prepared using standard metallographic procedures. Specimens were cut from the center by a precise grinding blade, ground with 1200-grit SiC paper, and then mechanically polished in an automatic vibrational polishing machine. Before oxidation experiments, initial microstructural characterizations were conducted using a HITACHI TM4000 plus tabletop microscope (Hitachi, Tokyo, Japan). The accelerating voltage was 15 kV and the working distance was 10 mm. After oxidation for 576 h, cross-sectional microstructures of oxide scales were characterized using a FEI quanta 650 scanning electron microscope (FEI Company, Eindhoven, The Netherlands) equipped with a secondary electron (SE) detector, a back-scattered electron (BSE) detector, and an energy dispersive X-ray spectroscopy (EDS) detector. The accelerating voltage was 10–20 kV and the working distance was 15 mm.

### 2.4. Thermodynamic Calculation

The phase diagram of Ni-15Al-Si and the activity of Al in Ni-15Al and Ni-15Al-2Si alloys in temperatures ranging from 700 °C to 1200 °C were calculated by Thermo-Calc software (version No. 2022.195234-411) using the TCNI9: Ni-alloys database (version No. 9.1).

## 3. Results

### 3.1. Initial Microstructures

**Figure 1:**
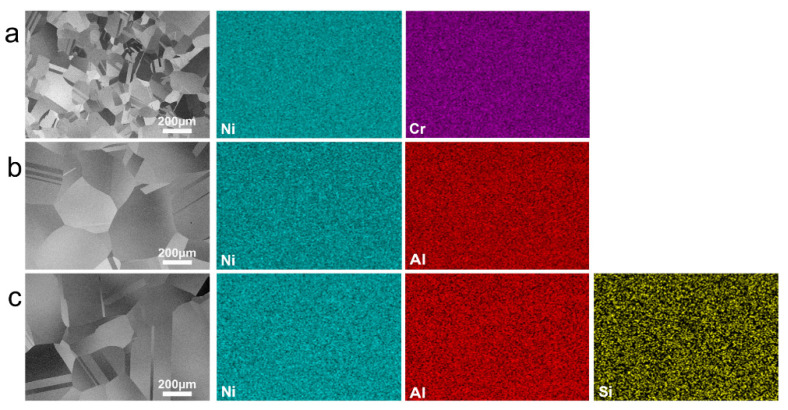
Initial microstructures (BSE images) and corresponding EDS maps of (**a**) Ni-20Cr, (**b**) Ni-15Al, and (**c**) Ni-15Al-2Si before oxidation experiments. Note that all three model alloys are Ni-based solid-solution alloys with uniformly distributed elements despite different grain sizes.

Figure 1 shows the representative microstructures and corresponding EDS maps of Ni-20Cr, Ni-15Al, and Ni-15Al-2Si alloys before oxidation experiments. After annealing at 1150 °C for 1 h, materials are fully recrystallized and contain uniform equiaxed grain microstructures with many annealing twins in grains. Determined by a linear intercept method, the average grain size without twin boundaries of Ni-20Cr (~61 µm) is relatively smaller than that of Ni-15Al (~228 µm) and Ni-15Al-2Si (~210 µm). The EDS maps in Figure 1 show that all elements distribute homogeneously, thus all three model alloys are Ni-based solid-solution alloys without intragranular or intergranular precipitates, which meets the initial experimental design.

### 3.2. Oxidation Kinetics Curves

**Figure 2:**
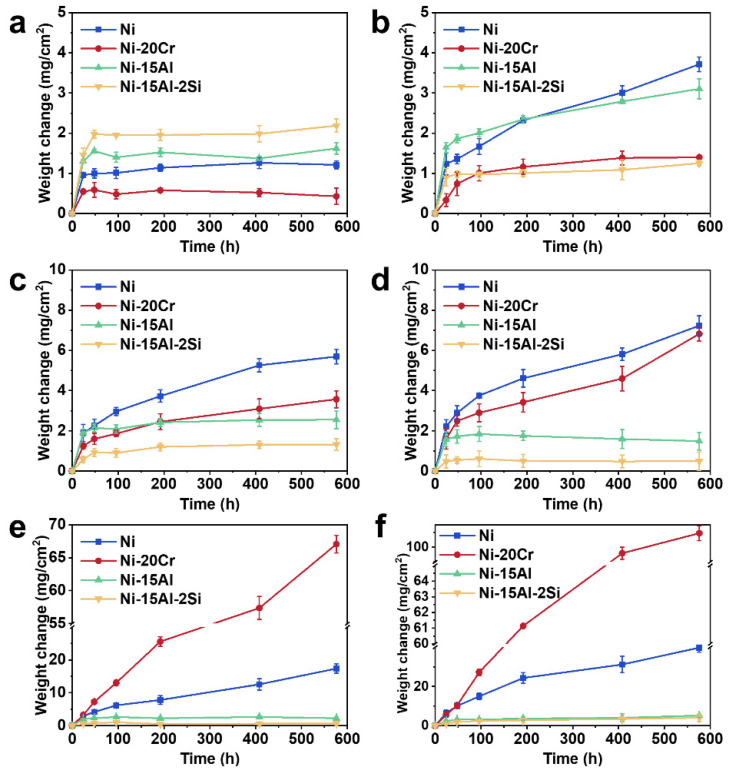
Oxidation kinetic curves of Ni, Ni-20Cr, Ni-15Al, and Ni-15Al-2Si alloys at (**a**) 700 °C, (**b**) 800 °C, (**c**) 900 °C, (**d**) 1000 °C, (**e**) 1100 °C, and (**f**) 1200 °C. Oxidation weight gains can directly reflect the oxidation performance of four samples.

Figure 2 shows the change on weight of Ni, Ni-20Cr, Ni-15Al, and Ni-15Al-2Si alloys with time at temperatures ranging from 700 °C to 1200 °C. Two prominent stages are observed for oxidation kinetic curves. At the initial stage (<100 h), the weight gains of four samples increase dramatically, indicating that alloys are fast oxidized. Upon extending the oxidation time, weight gains increase more slowly compared to the initial stage.

At 700 °C (Figure 2a), the final oxidation weight gains can be ranked from large to small as follows: Ni-15Al-2Si > Ni-15Al > Ni > Ni-20Cr. When the temperature rises to 800 °C (Figure 2b), the final weight gain of Ni increases to 3.7 mg/cm^2^ and surpasses that of Ni-15Al. The final weight gain of Ni-15Al-2Si perversely decreases to be smaller than that of Ni-20Cr, making the order of the final weight gains at 800 °C as follows: Ni > Ni-15Al > Ni-20Cr > Ni-15Al-2Si. From 900 °C to 1000 °C (Figure 2c,d), the final weight gain of Ni-20Cr exceeds that of Ni-15Al, becoming secondary among four samples and gradually approaching that of Ni. The final weight gain of Ni-15Al-2Si is always below 2 mg/cm^2^ and smaller than that of Ni-15Al. Another prominent change occurs at 1100 °C (Figure 2e). The weight gain of Ni-20Cr exceeds that of Ni and increases dramatically with the rising temperature. At 1200 °C (Figure 2f), the final weight gain of Ni-20Cr surprisingly reaches 109.4 mg/cm^2^, while that of Ni is only 39.8 mg/cm^2^. Meanwhile, the final weight gains of Ni-15Al and Ni-15Al-2Si are significantly smaller than that of Ni and Ni-20Cr, which are 4.4 mg/cm^2^ and 4.0 mg/cm^2^, respectively.

Using Ni as the criterion for comparison, it can be found that Cr, Al, and Si have distinct influences on the oxidation behaviors of Ni-based alloys. At 700 °C, Cr addition can improve the oxidation performance, while additions of Al and Si slightly aggravate oxidation. From 800 °C to 1000 °C, additions of Cr, Al, and Si are beneficial for the inhibition of oxidation. Above 1100 °C, the Cr addition induces much more severe oxidation of Ni-20Cr than other samples.

### 3.3. Cross-Sectional Microstructure and Composition of Oxide Scales

#### 3.3.1. Analysis of Ni, Ni-20Cr, and Ni-15Al Oxide Scales

**Figure 3:**
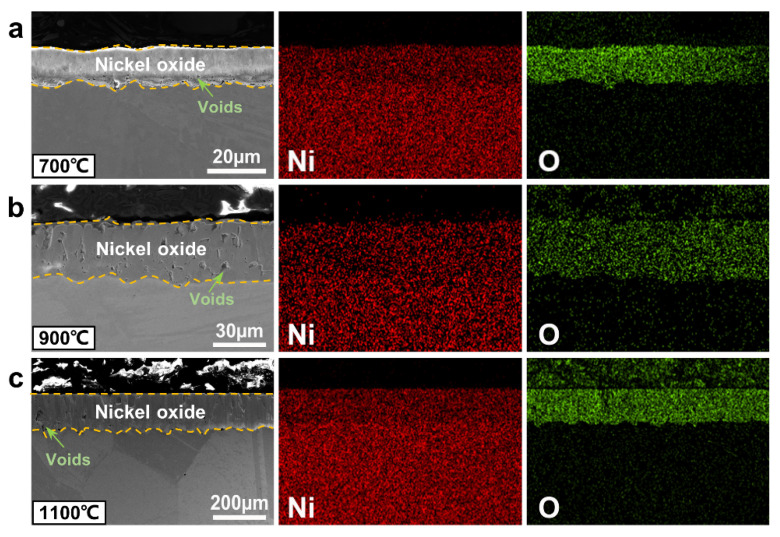
Cross-sectional microstructures (SE images) and corresponding EDS maps of oxide scales formed on Ni oxidized at (**a**) 700 °C, (**b**) 900 °C, and (**c**) 1100 °C for 576 h. Note that Ni forms a monolayer nickel oxide.

Cross-sectional microstructures and EDS maps of oxide scales on Ni after oxidation at a temperature range of 700 °C to 1200 °C for 576 h are shown in Figure 3 and Supplementary Figure S1. The oxide scale formed on Ni always exhibits a monolayer structure at different temperatures, which is nickel oxide according to the elemental composition of Ni and O in EDS maps. Voids in the nickel oxide layer (indicated by green arrows) demonstrate that this layer is loose and nonprotective to inhibit further oxidation. Moreover, the nickel oxide layer dramatically thickens from 12.2 µm to 336.9 µm, when the temperature increases from 700 °C to 1200 °C.

**Figure 4:**
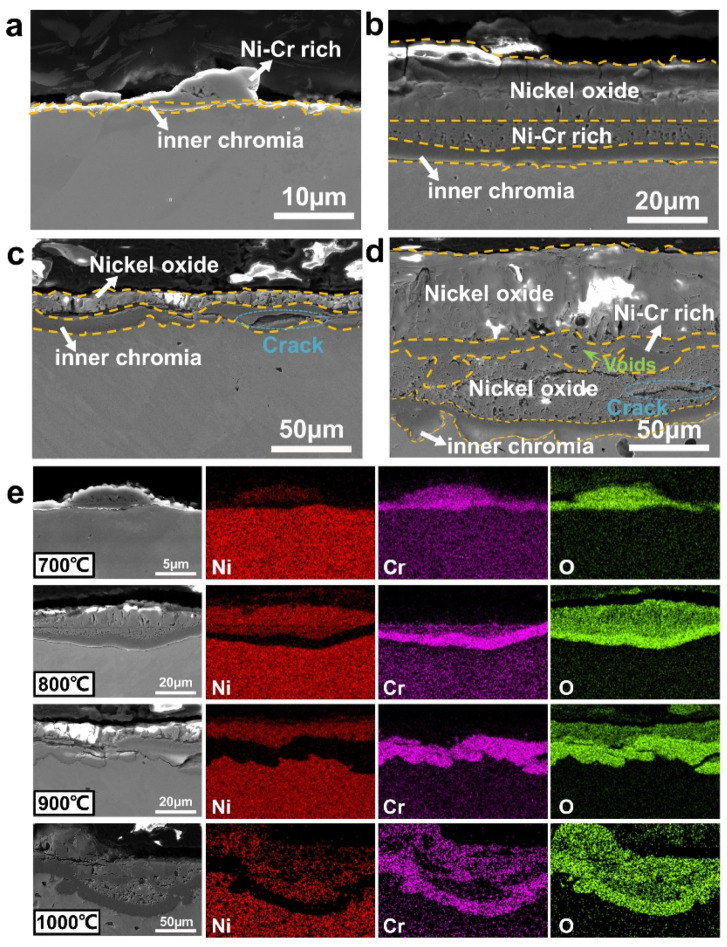
Cross-sectional microstructures (SE images) of oxide scales formed on Ni-20Cr alloy oxidized at (**a**) 700 °C, (**b**) 800 °C, (**c**) 900 °C, and (**d**) 1000 °C for 576 h. Note that the integrity of the chromia layer is destroyed with rising temperatures, which leads to the degradation of oxidation resistance. (**e**) EDS maps of oxide scales formed on Ni-20Cr alloy at temperatures ranging from 700 °C to 1000 °C.

Figure 4 shows cross-sectional microstructures and EDS maps of oxide scales on Ni-20Cr oxidized at temperatures ranging from 700 °C to 1000 °C. After oxidation at 700 °C for 576 h (Figure 4a), a thin oxide film and some island-like oxide protrusions are formed on Ni-20Cr. According to EDS maps (Figure 4e), the oxide film contains mainly chromia and the oxide protrusions are Ni-Cr rich oxides. As shown in Figure 4b, Ni-20Cr oxidized at 800 °C for 576 h forms a triplex oxide scale, which is composed of an external nickel oxide layer, an intermediate Ni-Cr rich layer, and an inner chromia layer. At 900 °C (Figure 4c,e), there is an external nickel oxide layer and an inner chromia layer overlaid on the surface of Ni-20Cr. Some microcracks (indicated by blue dotted ellipse) are observed at the alloy/scale interface, thus it is difficult to protect the base alloy with such oxide layer as a diffusion barrier. When the oxidation temperature reaches 1000 °C (Figure 4d,e), complicated multilayer oxide scales have been formed on Ni-20Cr. Between the external nickel oxide layer and inner chromia layer, discontinuous nickel oxides and Ni-Cr rich oxides are interlaced together with many microcracks (e.g., marked by blue dotted ellipse in Figure 4d) and voids (indicated by green arrows in Figure 4d) showing the occurrence of severe oxidation, which is consistent with the significant final weight gain of Ni-20Cr shown in Figure 2d. Microstructures of oxide scales on Ni-20Cr above 1100 °C (shown in Supplementary Figure S2) are similar to those at 1000 °C.

**Figure 5:**
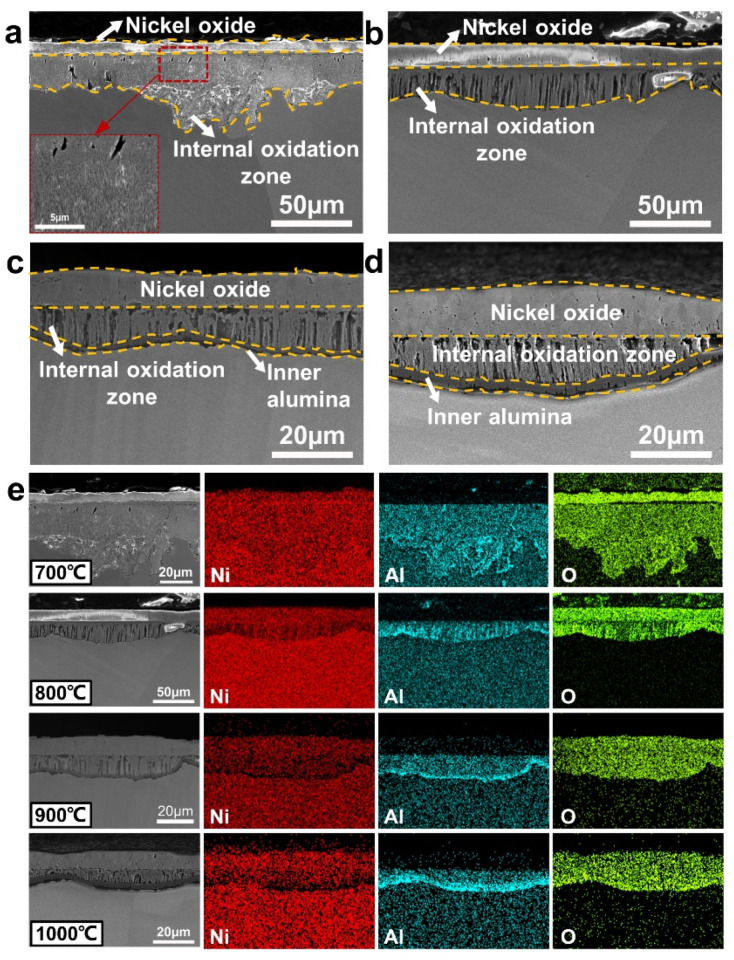
Cross-sectional microstructures (SE images) of oxide scales formed on Ni-15Al alloy oxidized at (**a**) 700 °C, (**b**) 800 °C, (**c**) 900 °C, and (**d**) 1000 °C for 576 h. Note that the formation of the alumina layer can effectively inhibit internal oxidation. (**e**) EDS maps of oxide scales formed on Ni-15Al alloy at temperatures ranging from 700 °C to 1000 °C.

Cross-sectional microstructures and EDS maps of oxide scales on Ni-15Al alloy at temperatures ranging from 700 °C to 1000 °C are displayed in Figure 5. After oxidation at 700 °C for 576 h (Figure 5a,e), Ni-15Al forms a duplex oxide scale composed of an external nickel oxide layer and an internal oxidation zone. Consistent with the results on oxidation of binary Ni-Al alloys [29,30,31,32], the internal oxidation zone contains many fine alumina oxides, which exhibit a closely spaced distribution, and acicular or granular morphologies (shown in the inset of Figure 5a). When the oxidation temperature rises higher, these alumina oxides in the internal oxidation zone become coarse to form a fiber-like morphology, which is perpendicular to the sample surface at 800 °C (Figure 5b) and a lumpy shape at 900 °C (Figure 5c). Moreover, in front of the internal oxidation zone, an inner alumina layer with a thickness of 1.3 µm is formed at 900 °C (Figure 5c) and thickens to 3.8 µm at 1000 °C (Figure 5d). After oxidation at 1100 °C for 576 h (Supplementary Figure S3a,c), with the complete avoidance of internal oxidation, Ni-15Al alloy is covered by a triplex oxide scale composed of a nickel oxide layer, an intermediate mixed oxide layer, and an inner alumina layer. When the temperature reaches 1200 °C (Supplementary Figure S3b,c), the external nickel oxide layer peels off to form a duplex oxide scale and the thickness of the inner alumina layer is increased to 15.3 µm.

Because oxide scales are not only a sheath separating the base alloy from oxygen but also oxide products, the oxidation resistance of alloys can be intuitively evaluated by comparing their thickness with that of Ni. Table 2 shows the thicknesses of oxide scales formed on Ni, Ni-20Cr, and Ni-15Al alloys oxidized at temperatures ranging from 700 °C to 1200 °C for 576 h. Below 900 °C, the thinner oxide scale on Ni-20Cr than that on Ni indicates that the addition of 20 at.% Cr can effectively inhibit oxidation. At temperatures above 1000 °C, thicknesses of oxide scales on Ni-20Cr increase rapidly with rising temperatures and are much larger than that on Ni, demonstrating that the addition of 20 at.% Cr is detrimental to oxidation resistance. At 700 °C and 800 °C, the addition of 15 at.% Al leads to aggravated oxidation according to the thickest oxide scales of Ni-15Al among the three samples. Meanwhile, at temperatures ranging from 900 °C to 1200 °C, the oxide scale on Ni-15Al is always thinner than that on Ni, showing improved oxidation resistance.

#### 3.3.2. Analysis of Ni-15Al-2Si Oxide Scales

**Figure 6:**
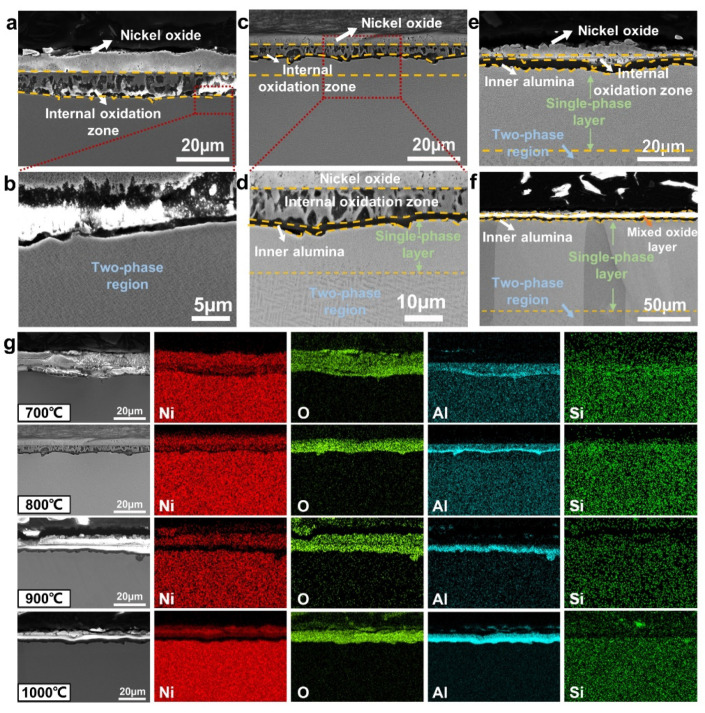
Cross-sectional microstructure (SE images) of oxide scales on Ni-15Al-2Si alloy oxidized at various temperatures for 576 h. (**a**,**b**) 700 °C; (**c**,**d**) 800 °C; (**e**) 900 °C; (**f**) 1000 °C. (**g**) EDS maps of oxide scales on Ni-15Al-2Si at temperatures ranging from 700 °C to 1000 °C.

Cross-sectional microstructures and EDS maps of Ni-15Al-2Si alloy at temperatures ranging from 700 °C to 1000 °C are shown in Figure 6. After oxidation at 700 °C for 576 h (Figure 6a,b), there is an external nickel oxide layer followed by an internal oxidation zone on Ni-15Al-2Si, which is similar to the oxide structure on Ni-15Al at 700 °C (Figure 5a). At 800 °C (Figure 6c,d), the front of the internal oxidation zone forms an inner oxide layer containing mainly alumina according to the EDS maps. When the temperature reaches 900 °C (Figure 6e), the depth of the internal oxidation zone decreases with a relatively thick alumina layer, indicating that the alumina layer can effectively prevent oxidation. At 1000 °C (Figure 6f,g), with the internal oxidation completely avoided, a mixed oxide layer, which contains Al, O, as well as a small amount of Ni and Si, is located between the external and inner layers. The external nickel oxide layer is partially peeled off and no longer continuous. Moreover, a certain amount of Si exists in the internal oxidation zone and mixed oxide layer (Figure 6g), indicating the formation of some Si-contained oxides.

Besides the variation in oxide scales with rising temperature, there are two additional noteworthy microstructural changes. First, at temperatures ranging from 700 °C to 900 °C, the coarsening phenomenon of alumina oxides in the internal oxidation zone also occurs with Si addition. At 700 °C, alumina oxides in Ni-15Al-2Si exhibit a lumpy morphology (Figure 6a), which is coarse with comparison to the rod-like morphology in Ni-15Al (Figure 5a). The coarser alumina oxides also contribute to the larger weight gain of Ni-15Al-2Si than that of Ni-15Al at 700 °C (Figure 1a), although the oxide scale of Ni-15Al-2Si is thinner than that of Ni-15Al. At 800 °C, alumina oxides in Ni-15Al-2Si merge into a thin alumina film in the front of the internal oxide zone (Figure 6c,d), while alumina oxides in Ni-15Al display a fine, fiber-like morphology (Figure 4b). When the temperature reaches 900 °C (Figure 6c), the thickness of the inner alumina layer of Ni-15Al-2Si reaches 1.8 µm and is much larger than that of Ni-15Al (0.5 µm). Therefore, Si addition can accelerate the formation of alumina in Ni-Al alloys during oxidation.

In addition, although the initial alloy is a single-phase supersaturated solid-solution, the long-term oxidation at temperatures ranging from 700 °C to 1000 °C for 576 h is also an aging (annealing) process, so the second phase precipitates and grows to form a two-phase structure in Ni-15Al-2Si as shown in Figure 6a–f. The thermodynamic equilibrium phase diagram of Ni-15Al-2Si calculated by Thermo-Calc software shown in Supplementary Figure S4 demonstrates that there are two equilibrium phases below 1100 °C. According to the calculated results, the matrix phase is solid-solution γ-Ni and the precipitated phase is γ’-Ni_3_Al. Meanwhile, selective oxidation consumes a large amount of Al and Si elements in the region close to oxide scales. Due to Al and Si depletion, this region cannot precipitate the γ’-Ni_3_Al phase, and is still in a solid-solution state, indicated as a single-phase layer in Figure 6d–f, which also can be found during the oxidation of other Ni-Al-Si alloys [31,32].

**Figure 7:**
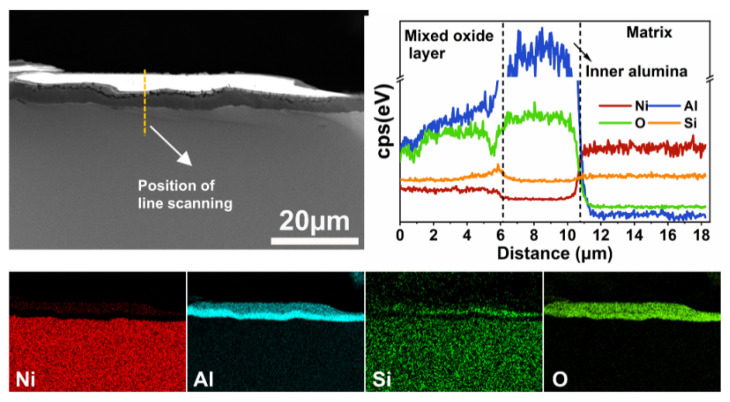
Cross-sectional microstructure (SE images), corresponding EDS maps, and line analysis of oxide scale on Ni-15Al-2Si alloy oxidized at 1100 °C for 576 h. Note that Si element is slightly enriched in the mixed oxide layer.

After oxidation at 1100 °C for 576 h (Figure 7), Ni-15Al-2Si alloy forms a duplex oxide scale, which is composed of a mixed oxide layer and an inner alumina layer. According to the EDS maps and line scanning in Figure 7, it can be found that Si is slightly enriched in the mixed oxide layer and is barely contained in the inner alumina layer. The bright contrast of the mixed oxide layer is caused by secondary electron charging in SEM. When the temperature rises to 1200 °C (Supplementary Figure S5b), only an alumina layer forms on Ni-15Al-2Si after oxidation for 576 h.

**Figure 8:**
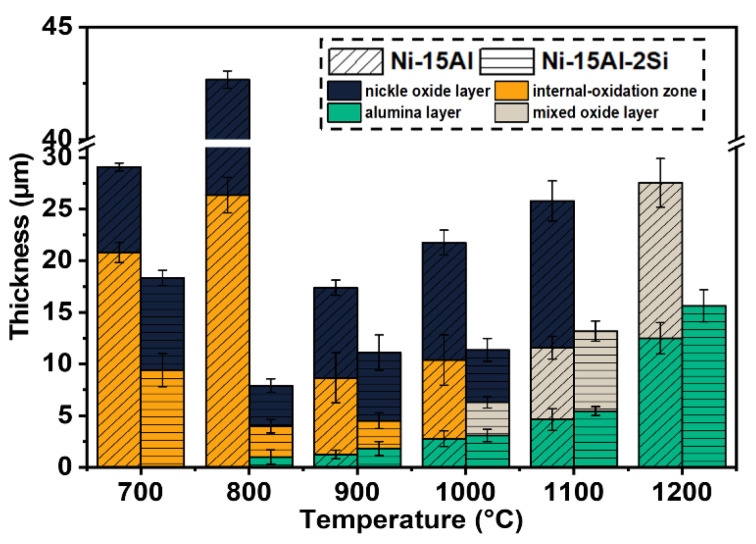
Effect of temperature on the type and thickness of oxide layers formed on Ni-15Al and Ni-15Al-2Si. Note that the formation of inner alumina layer is crucial to excellent oxidation resistance of the Ni-Al alloy. Si addition can accelerate the formation of inner alumina layer.

Figure 8 shows the type and thickness of oxide layers on Ni-15Al and Ni-15Al-2Si alloys oxidized at temperatures ranging from 700 °C to 1200 °C for 576 h. It can be found that the formation of the inner alumina layer is crucial to the oxidation behaviors of Ni-Al alloys. In the absence of the alumina layer, the occurrence of internal oxidation will be inevitable and an oxide scale with a large thickness will form on the alloy. The addition of Si reduces the forming temperature of the inner alumina layer in Ni-Al alloys from 900 °C to 800 °C. In the temperature range of 900 °C to 1200 °C, the thickness of the alumina layer on Ni-15Al-2Si oxidized at the same temperature for 576 h is always larger than that on Ni-15Al. It once again demonstrates that the addition of 2 at.% Si can form a synergistic effect with Al to accelerate the formation of alumina.

## 4. Discussion

### 4.1. Oxidation Kinetics

Generally, the relationship between weight gain and oxidation time can be described as a power law, which is often used to understand oxidation kinetics [33,34,35,36]:

(1) $$\Delta w=kt^{n}+C$$

where Δ*w* is the mass change per unit area, *k* corresponds to an oxidation rate constant, *t* is the exposure time, *n* represents the oxidation mass gain reaction index, and *C* is a constant, which is equal to zero in current experimental conditions.

By fitting experimental data to Equation (1), the value of *n* and oxidation rate constant *k* can be obtained. Because the change in oxidation weight of four samples at 700 °C and 800 °C is smaller than the measurement error of the electronic analytical balance, only oxidation kinetic data at temperatures ranging from 900 °C to 1200 °C is analyzed in this section.

According to the oxidation theory of Wanger et al. [2,5], the specific oxidation process can be identified by the distinct *n* value. Except for the oxidation of Ni-20Cr at 1100 °C and 1200 °C, *n* values of all samples at temperatures ranging from 900 °C to 1200 °C are close to 0.5, which indicates that the oxidation kinetics obeys a parabolic law and the oxidation process is controlled by ionic diffusion through oxide scales. The oxidation of Ni-20Cr at 1100 °C and 1200 °C obeys the level linear law due to the *n* values close to 1.0. In this case, the gas oxide interface reaction dominates the oxidation process, and the activity of metal ions at the interface remains at a relatively high level [5,36].

To compare oxidation behaviors with other alloy systems, *k* values are calculated by using the data fitted with *n* equal to 0.5. The results are shown in Table 3. At 900 °C and 1000 °C, calculated *k* values are following the order Ni > Ni-20Cr > Ni-15Al > Ni-15Al-2Si, which demonstrates that additions of Cr, Al, and Si all can reduce the oxidation rate. The *k* value generally increases with increasing temperature, and at 1100 and 1200 °C, *k* values of Ni-20Cr increase to 9.8 × 10^−4^ and 6.2 × 10^−3^ [mg^2^/(cm^4^s^2^)], respectively. *k* values of Ni-15Al and Ni-15Al-2Si are found between 3.1 × 10^−6^ and 2.1 × 10^−5^ [mg^2^/(cm^4^s^2^)], showing improved oxidation resistance after additions of Al and Si.

The oxidation rate constant *k* is related to the oxidation activation energy, which can be expressed by the Arrhenius equation:

(2) k=k0exp−QRT

where *k* is the oxidation rate constant, *k*_0_ is a constant for the equation, *Q* is the activation energy, *R* is the molar gas constant (8.3144 J/K mol), and *T* is the absolute temperature. 

**Figure 9:**
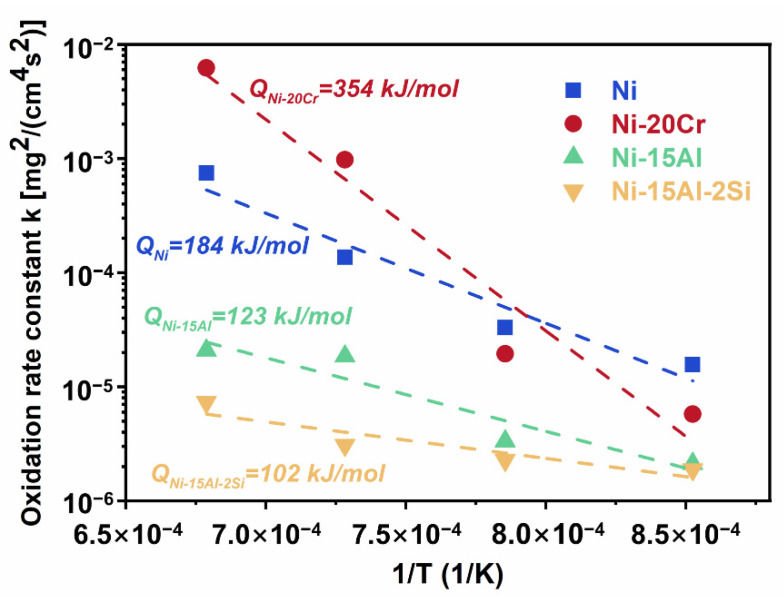
Temperature dependence of oxidation rate constants of Ni, Ni-20Cr, Ni-15Al, and Ni-15Al-2Si alloys at temperatures ranging from 900 °C to 1200 °C. Slopes of curves represent activation energy *Q* in Equation (2).

Activation energies are calculated by linear fitting of the ln(*k*) vs. 1/*T* plot, as shown in Figure 9. It can be seen that the additions of Cr, Al, and Si have significant effects on the oxidation activation energy. The calculated oxidation activation energy of pure Ni is 184 kJ/mol, which is in good agreement with the calculation by Douglass (188 kJ/mol) [37]. Compared with the reported value of a Ni-10Cr alloy (170 kJ/mol) [37], Ni-20Cr alloy has a relatively high oxidation activation energy (354 kJ/mol), which may be caused by the severely volatile chromium oxides above 1000 °C. The activation energy of Ni-15Al is 123 kJ/mol, which is slightly different from the reported value of Al oxidation (96 kJ/mol) [38]. This slight deviation might result from the simultaneous generation of nickel oxide and alumina. The activation energy of Ni-15Al (123 kJ/mol) is close to that of Al oxidation (96 kJ/mol), significantly different from that of Ni oxidation (188 kJ/mol), indicating that Al dominates the oxidation process. Further reduction of activation energy (102 kJ/mol) after adding 2 at.% Si to Ni-15Al alloy indicates that Si addition can promote the formation of alumina.

### 4.2. Oxidation Mechanism and Oxide Layer Formation

There are mainly three types of oxide layers formed on Ni-20Cr, including the external nickel oxide layer, the Ni-Cr rich layer, and the inner chromia layer. According to the XRD pattern of oxides on Ni-20Cr alloy (shown in Supplementary Figure S6), the nickel oxide layer should be composed of NiO, and the main oxide phase of the inner chromia layer is Cr_2_O_3_. The Ni-Cr rich layer may consist of NiCr_2_O_4_ or a mixture of NiO, NiCr_2_O_4_, and Cr_2_O_3_. The NiCr_2_O_4_ spinel can be formed as:

(3) $${\mathrm{Cr}}_{2}O_{3}+\mathrm{NiO}={\mathrm{NiCr}}_{2}O_{4}$$

If the diffusion of oxygen ions into the alloy is fast, NiCr_2_O_4_ spinel can also be formed as:

(4) Cr2O3+Ni+12O2= NiCr2O4

When the temperature exceeds 1000 °C, the integrity of oxide scales can be destroyed by the formation of volatile CrO_3_ [5,6,7,13,15]:

(5) Cr2O3+32O2=2CrO3

The base alloy is exposed to the air and rapidly oxidized at a high temperature. At temperatures above 1000 °C, the repeating process of “germinating—growing—spalling—germinating” leads to high weight gain and the complex “NiO—NiCr_2_O_4_” multilayer oxide structure for Ni-20Cr alloy, as shown in Figure 4d and Supplementary Figure S2.

**Figure 10:**
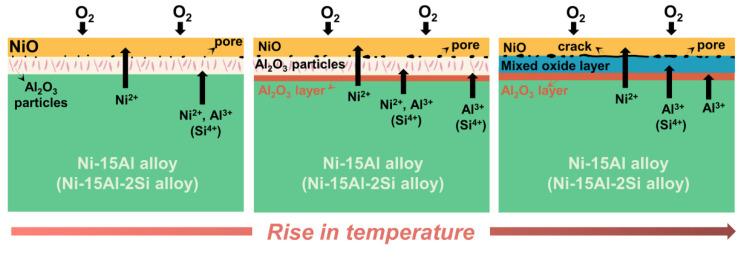
Mechanism diagram showing the influence of temperature on the evolution of oxide scales on Ni-15Al and Ni-15Al-2Si alloys oxidized in a temperature range of 700 °C to 1200 °C.

According to XRD patterns (Supplementary Figure S6), oxides formed on Ni-15Al mainly consist of NiO, NiAl_2_O_4_, and Al_2_O_3_. In addition to the above three oxide phases, Ni_2_SiO_4_ may also exist in oxide scales of Ni-15Al-2Si. It can be inferred that the nickel oxide layer contains mainly NiO and the inner alumina is an Al_2_O_3_ layer. Combined with oxide microstructures and XRD patterns of oxide phases, their detailed oxidation mechanisms are summarized and depicted in a schematic illustration, as shown in Figure 10.

Both Ni-15Al and Ni-15Al-2Si alloys form an internal oxidation zone after oxidation at relatively low temperatures for 576 h. There are many acicular or rod-like Al_2_O_3_ particles dispersed in this zone (the left image of Figure 10). With the rising temperature, more Al_2_O_3_ is generated and merges into an Al_2_O_3_ layer at the front of the internal oxidation zone (the middle image of Figure 10). In the presence of the Al_2_O_3_ layer, the internal diffusion of oxygen ions is effectively inhibited. When the temperature rises above 1100 °C, the internal oxidation zone no longer exists and a mixed oxide layer, possibly composed of NiAl_2_O_4_ spinel, is formed between the external and internal layers (the right image of Figure 10).

There are still some differences in the evolution of oxide scales on Ni-15Al and Ni-15Al-2Si. The addition of 2 at.% Si reduces the forming temperature of the Al_2_O_3_ layer from 900 °C to 800 °C. In addition, there is no internal oxidation zone observed on Ni-15Al-2Si after oxidation at 1000 °C for 576 h. The thickness of the Al_2_O_3_ layer oxidized on Ni-15Al-2Si is always larger than that on Ni-15Al at the same temperature. It can be inferred that the addition of 2 at.% Si can accelerate the formation of Al_2_O_3_ in Ni-Al alloys. As reported by Yeh et.al [27,28], the expedited formation of Al_2_O_3_ after Si addition is attributed to the increasing activity of Al. Supplementary Table S1 displays the activity of Al in Ni-15Al and Ni-15Al-2Si at temperatures ranging from 700 °C to 1200 °C, which is calculated by the Thermo-Calc software using the TCNI9 database. Consistent with experimental results, the activity of Al in Ni-15Al-2Si is always higher than that in Ni-15Al at the same temperature.

## 5. Summary and Conclusions

To reveal the effects of Cr, Al, and Si on the oxidation resistance of Ni-based superalloys, isothermal oxidation behaviors of three model Ni-based alloys (Ni-20Cr, Ni-15Al, and Ni-15Al-2Si) and pure Ni at temperatures ranging from 700 °C to 1200 °C were investigated. Results demonstrate that additions of Cr, Al, and Si have distinct effects on the oxidation resistance of Ni-based alloys at different temperatures. Based on the oxidation test and subsequent microstructural analyses, the following conclusions can be drawn:

1. The addition of 20 at.% Cr can effectively improve the oxidation resistance of Ni-based alloy by forming a thin chromia film below 900 °C. However, the protection of chromia is reduced due to formation of volatile CrO_3_ above 1000 °C, resulting in catastrophic oxidation.
2. The addition of 15 at.% Al can form an inner alumina layer above 900 °C to provide effective protection, thus showing excellent oxidation resistance at higher temperatures. However, at temperatures below 800 °C, Al addition intensifies oxidation due to the high oxygen affinity of Al, and continuous protective scales cannot form.
3. At 900 °C, the additions of Al and Cr can both improve the oxidation resistance, while the effect of Al on oxidation resistance is more pronounced than that of Cr.
4. Si can form a synergistic effect with Al to affect the oxidation resistance, rather than forming a silicon oxide layer at temperatures between 700 °C and 1200 °C. By promoting alumina formation, the addition of 2 at.% Si can improve the oxidation resistance of Ni-Al alloys above 800 °C.
5. At the relatively low temperature of 700 °C, the addition of Si cannot promote the formation of the protective alumina layer in Ni-Al alloy, where coarse alumina particles in the internal oxidation zone are formed.

## Figures and Tables

**Table 1:** Nominal composition of pure Ni and three model alloys (at.%).

Samples	Ni	Cr	Al	Si
Ni	100.0	-	-	-
Ni-20Cr	80.0	20.0	-	-
Ni-15Al	85.0	-	15.0	-
Ni-15Al-2Si	83.0	-	15.0	2.0

**Table 2:** Thicknesses of oxide scales (µm) on Ni, Ni-20Cr, and Ni-15Al alloys after oxidation at temperatures ranging from 700 °C to 1200 °C for 576 h.

Temperature	Ni	Ni-20Cr	Ni-15Al
700 °C	12 (±1)	2.2 (±0.8)	29.1 (±1.4)
800 °C	40 (±3)	26.1 (±2.1)	42.7 (±2.1)
900 °C	48.7 (±4.9)	20.7 (±2.3)	18.9 (±4.2)
1000 °C	51.6 (±3.2)	107.9 (±11.0)	22.7 (±4.4)
1100 °C	141.7 (±11.0)	>300	26.9 (±3.2)
1200 °C	336.9 (±57.2)	>400	27.6 (±3.7)

**Table 3:** Calculated *k* values [mg^2^/(cm^4^s^2^)] of Ni, Ni-20Cr, Ni-15Al, and Ni-15Al-2Si alloys at temperatures ranging from 900 °C to 1200 °C.

Temperatures	Ni	Ni-20Cr	Ni-15Al	Ni-15Al-2Si
900 °C	2.1 × 10^−5^	5.8 × 10^−6^	2.1 × 10^−6^	1.9 × 10^−6^
1000 °C	4.4 × 10^−5^	2.0 × 10^−5^	3.3 × 10^−6^	2.3 × 10^−6^
1100 °C	9.0 × 10^−5^	9.8 × 10^−4^	1.9 × 10^−5^	3.1 × 10^−6^
1200 °C	6.6 × 10^−4^	6.2 × 10^−3^	2.1 × 10^−5^	7.3 × 10^−6^

## Data Availability

The data presented in this study are available on request from the corresponding author.

## Supplementary Materials

The following supporting information can be downloaded at: https://www.mdpi.com/article/10.3390/ma15207352/s1, Figure S1: Cross-sectional microstructures and EDS maps of oxide scales formed on Ni at 800 °C, 1000 °C and 1200 °C for 576 h; Figure S2: Cross-sectional microstructures and EDS maps of oxide scales formed on Ni-20Cr at 1100 °C and 1200 °C for 576 h; Figure S3: Cross-sectional microstructures and EDS maps of oxide scales formed on Ni-15Al at 1100 °C and 1200 °C for 576 h; Figure S4: Thermodynamic equilibrium phase diagram of Ni-15Al-2Si calculated by Thermo-Calc software; Figure S5: Cross-sectional microstructures and EDS maps of oxide scales formed on Ni-15Al-2Si at 1100 °C and 1200 °C for 576 h; Figure S6: Representative XRD patterns of oxides formed on Ni-20Cr, Ni-15Al, and Ni-15Al-2Si after 576 h oxidation at 1000 °C; Table S1: The activity of Al in Ni-15Al and Ni-15Al-2Si calculated by Thermo-Calc software.
