# Supplementary material for: The Effects of Alloying Elements Cr, Al, and Si on Oxidation Behaviors of Ni-Based Superalloys
Source: Materials (Basel). 2022 Oct 20;15(20):7352. doi: 10.3390/ma15207352 (PMC9609945; doi:10.3390/ma15207352)
Supplement: Supplementary file 1 [file materials-15-07352-s001.zip › materials-1965615-supplementary.pdf]

Supplementary Information

for

The effects of alloying elements Cr, Al, and Si on oxidation behaviors of Ni-based superalloys

Suyu Ma[1,2], Qingqing Ding[1,*], Xiao Wei[1], Ze Zhang[1], Hongbin Bei[1,*]

1 *School of Materials Science and Engineering, Zhejiang University, Hangzhou, 310027, China*

2 *Polytechnic Institute, Zhejiang University, Hangzhou 310027, China;*

*Corresponding author: qq_ding@zju.edu.cn (Q. Ding); hbei2018@zju.edu.cn (H. Bei)

This document contains six supplementary figures and one supplementary table.

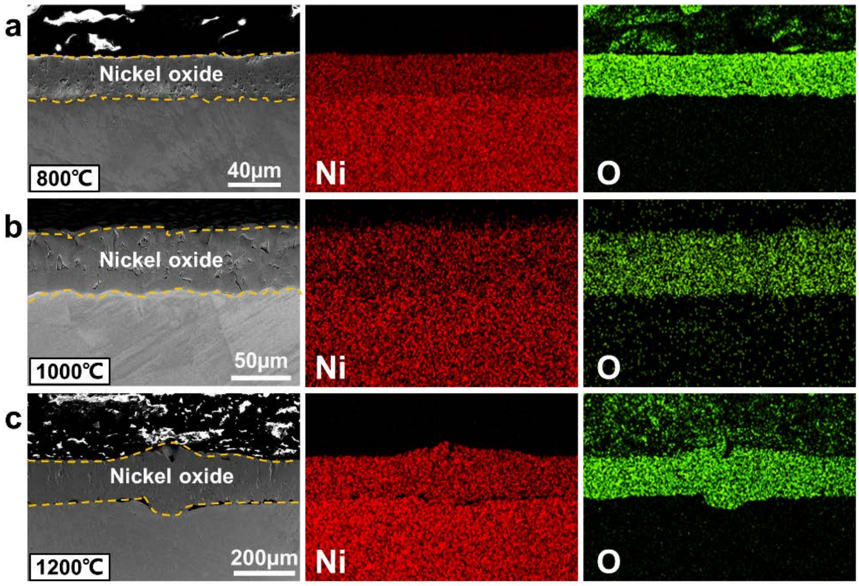

Figure S1. Cross-sectional microstructures (SE images) and EDS maps of oxide scales on Ni oxidized at (a) 800 °C, (b) 1000 °C, and (c) 1200 °C for 576 h.

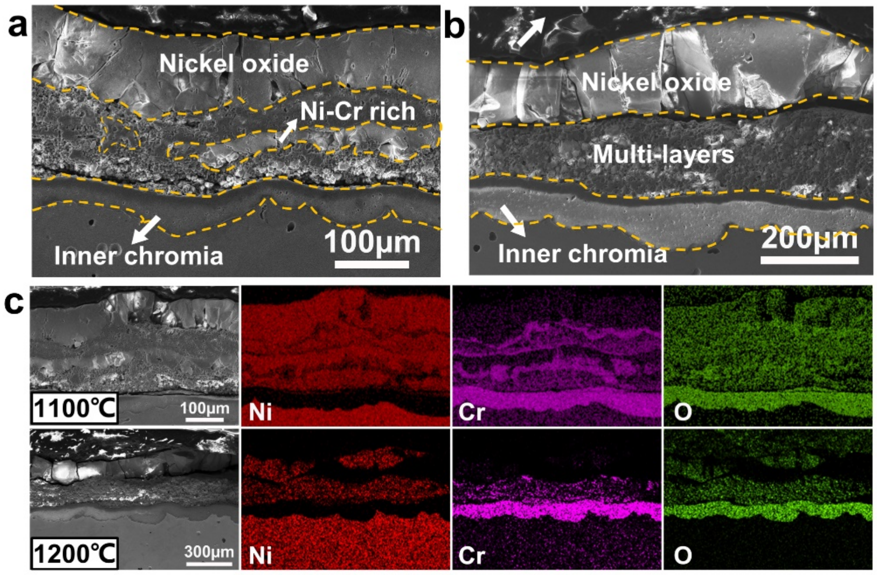

Figure S2. Cross-sectional microstructures (SE images) of oxide scales on Ni-20Cr oxidized at (a) 1100 °C, and (b) 1200 °C for 576 h. (c) EDS maps of oxide scales on Ni-20Cr oxidized at 1100 °C and 1200 °C for 576 h.

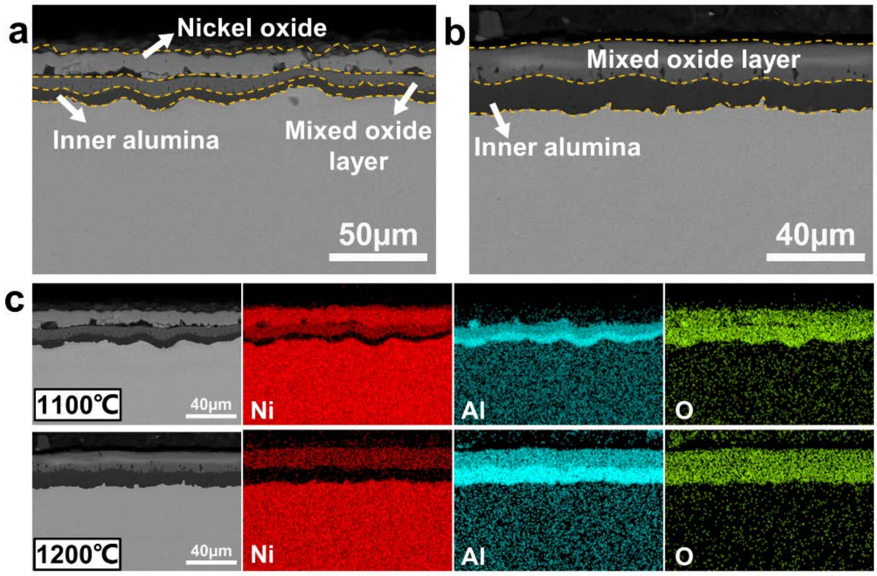

Figure S3. Cross-sectional microstructures (BSE images) of oxide scales on Ni-15Al oxidized at (a) 1100 °C, and (b) 1200 °C for 576 h. (c) EDS maps of oxide scales on Ni-15Al oxidized at 1100 °C and 1200 °C for 576 h.

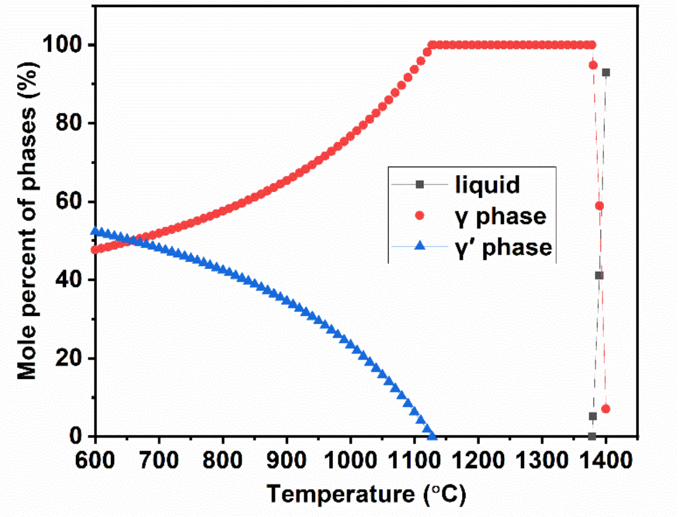

Figure S4. Thermodynamic equilibrium phase diagram of Ni-15Al-2Si calculated by Thermo-Calc software.

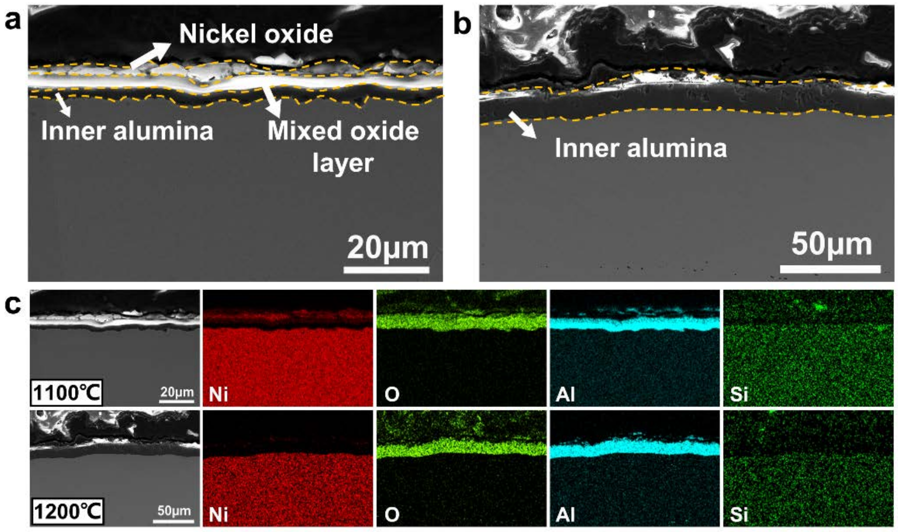

Figure S5. Cross-sectional microstructures (SE images) of oxide scales on Ni-15Al-2Si oxidized at (a) 1100 °C, and (b) 1200 °C for 576 h. (c) EDS maps of oxide scales on Ni-15Al-2Si oxidized at 1100 °C and 1200 °C for 576 h.

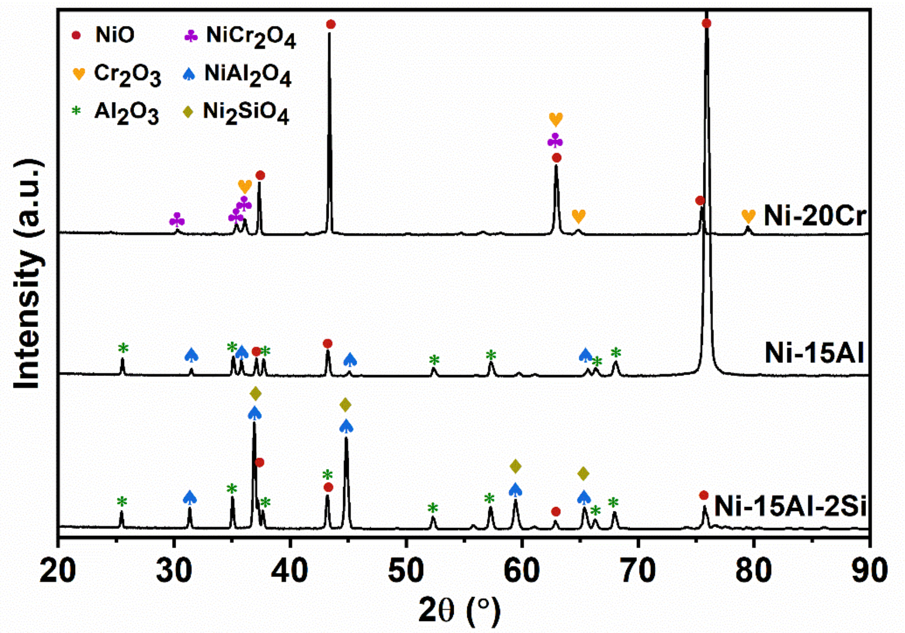

Figure S6. XRD patterns of oxides formed on Ni-20Cr, Ni-15Al, and Ni-15Al-2Si oxidized at 1000 °C for 576 h.

Table S1: Activity of Al in Ni-15Al and Ni-15Al-2Si at temperatures ranging from 700 °C to 1200 °C calculated by the Thermo-Calc software.

Temperatures	The activity of Al in Ni-15Al	The activity of Al in Ni-15Al-2Si
700 °C	1.71×10^{-10}	1.87×10^{-10}
800 °C	1.07×10^{-9}	1.17×10^{-9}
900 °C	4.84×10^{-9}	5.34×10^{-9}
1000 °C	1.44×10^{-8}	1.91×10^{-8}
1100 °C	3.04×10^{-8}	5.34×10^{-8}
1200 °C	5.69×10^{-8}	9.58×10^{-8}
